# Supplementary material for: Non-Pathogenic Mopeia Virus Induces More Robust Activation of Plasmacytoid Dendritic Cells than Lassa Virus
Source: Viruses. 2019 Mar 21;11(3):287. doi: 10.3390/v11030287 (PMC6466290; doi:10.3390/v11030287)
Supplement: Supplementary file 1 [file viruses-11-00287-s001.pdf]

Figure S1

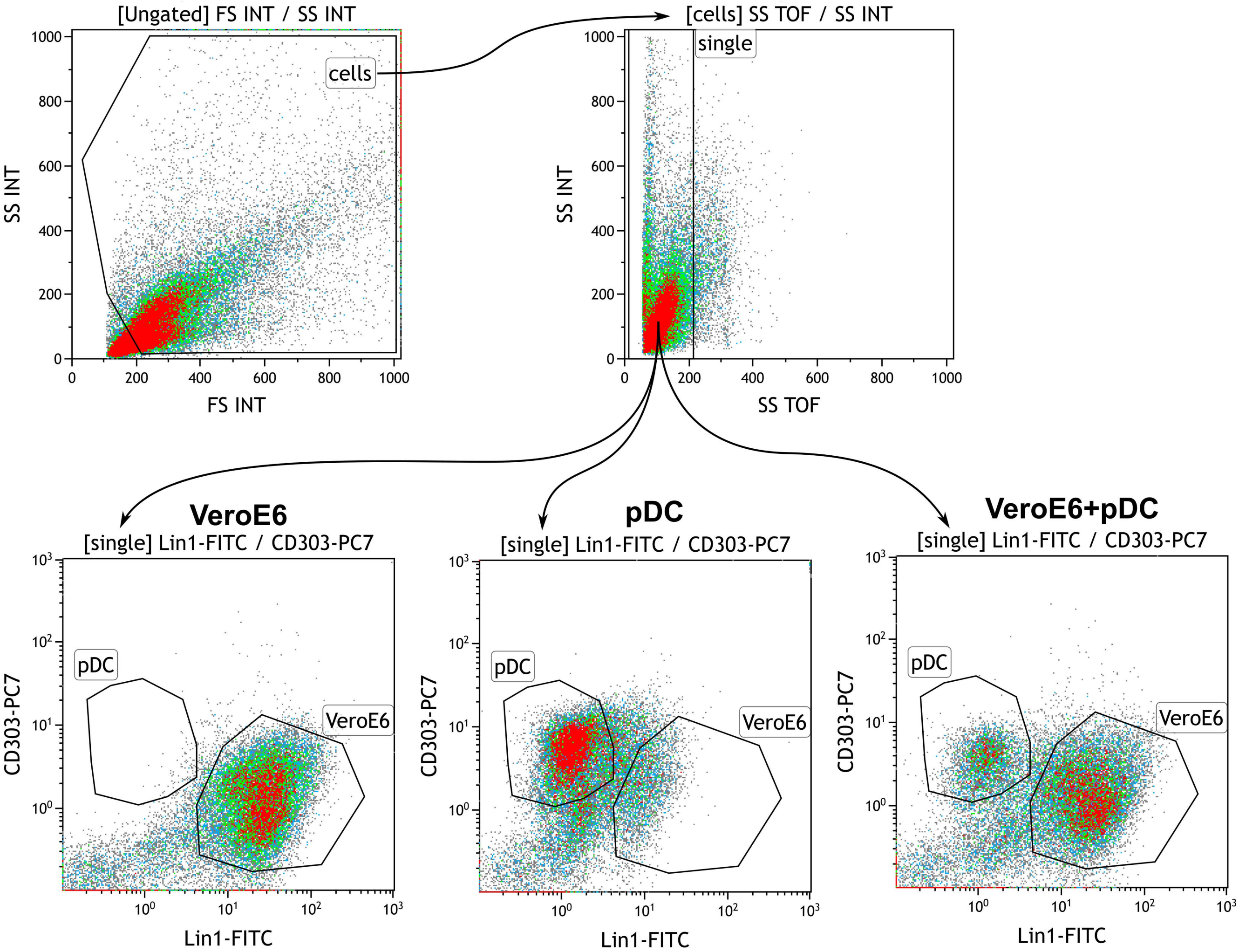

[Ungated] FS INT / SS INT
cells
SS INT
FS INT
0
200
400
600
800
1000
[cells] SS TOF / SS INT
single
SS TOF
VeroE6
[single] Lin1-FITC / CD303-PC7
pDC
VeroE6
CD303-PC7
Lin1-FITC
pDC
[single] Lin1-FITC / CD303-PC7
VeroE6+pDC
[single] Lin1-FITC / CD303-PC7

Figure S2

Analytes	No condition above threshold	Inside range but no significant differences	significant differences between at least 2 conditions
EGF		x	
Eotaxin		x	
FGF-2	x		
Flt-3L	x		
Fractalkine	x		
G-CSF		x	
GM-CSF		x	
GRO		x	
IFNα2			x
IFNγ		x	
IL-10		x	
IL12-p40		x	
IL12-p70		x	
IL-13	x		
IL-15			x
IL-17A	x		
IL-1α		x	
IL-1β		x	
IL-1RA		x	
IL-2	x		
IL-3	x		
IL-4	x		
IL-5		x	
IL-6			x
IL-7	x		
IL-8		x	
IL-9	x		
IP-10			x
MCP-1			x
MCP-3		x	
MDC		x	
MIP-1α		x	
MIP-1β			x
sCD40L		x	
TGFα		x	
TNFα			x
TNFβ			x
VEGF		x	
MPIF-1		x	
BRAK	x		
CXCL16		x	
HCC-4	x		
MIP-4	x		
IL-34		x	
IL-24		x	
APRIL		x	
IL-35	x		
IL-37	x		
IL-19		x	
CCL28	x		
HMGB1	x		
IFNβ			x
IL-38	x		
IL28B		x	
BAFF	x		
IL-14		x	
IL-36β	x		
IL32α		x	
YKL40	x		
IL-2	x		

Figure S3

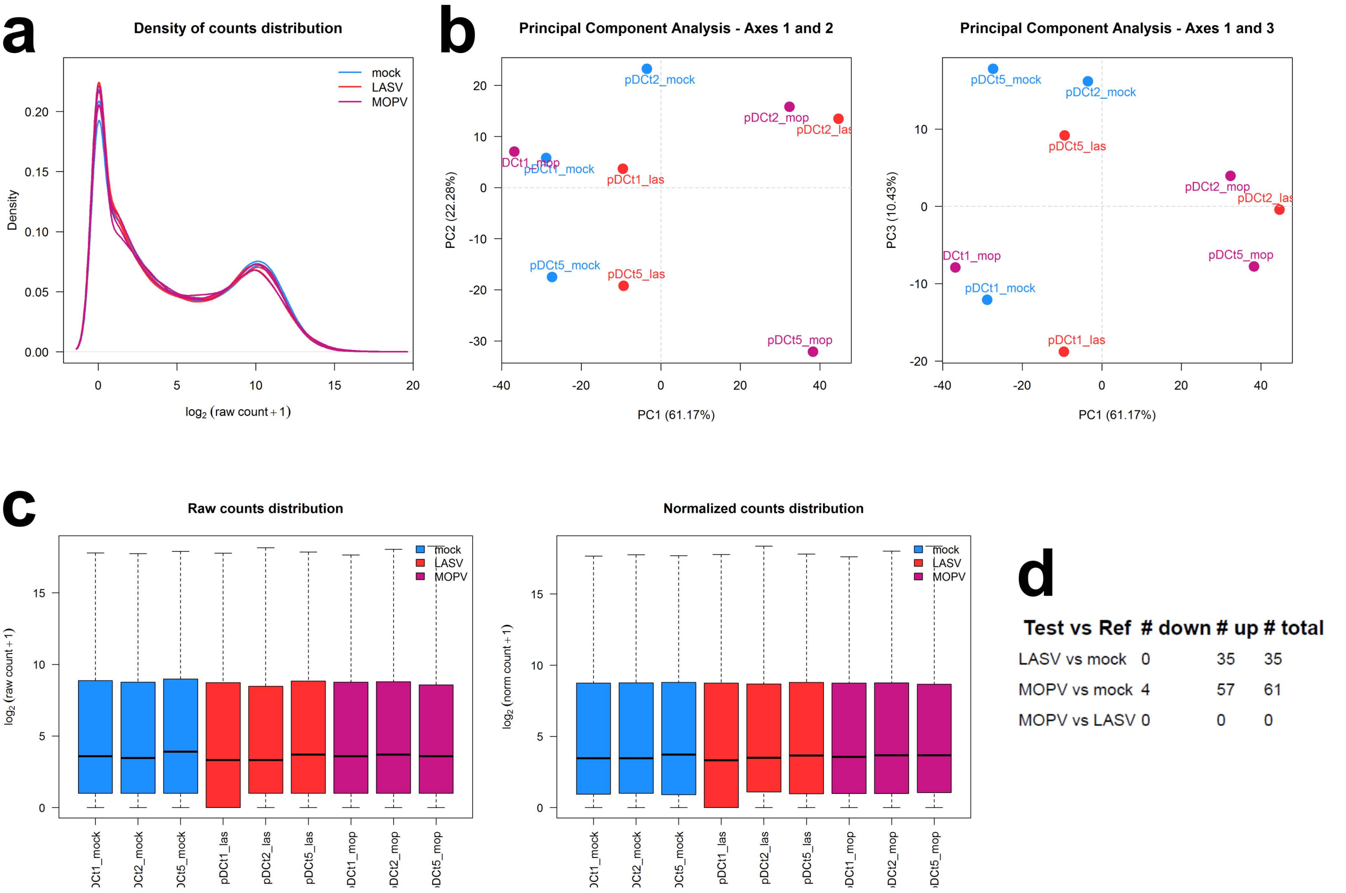

d

Test vs Ref	# down	# up	# total
LASV vs mock	0	35	35
MOPV vs mock	4	57	61
MOPV vs LASV	0	0	0
